# Course of postoperative relapse in non‐small cell lung cancer is strongly associated with post‐progression survival

**DOI:** 10.1111/1759-7714.14119

**Published:** 2021-09-03

**Authors:** Hisao Imai, Ryoichi Onozato, Kyoichi Kaira, Sayaka Kawashima, Ken Masubuchi, Toshiteru Nagashima, Kohei Tajima, Koichi Minato

**Affiliations:** ^1^ Division of Respiratory Medicine Gunma Prefectural Cancer Center Ota Japan; ^2^ Department of Respiratory Medicine Comprehensive Cancer Center, International Medical Center, Saitama Medical University Hidaka Japan; ^3^ Division of Thoracic Surgery Gunma Prefectural Cancer Center Ota Japan; ^4^ Division of Pharmacy Gunma Prefectural Cancer Center Ota Gunma Japan

**Keywords:** non‐small cell lung cancer, overall survival, postoperative recurrence, post‐progression survival, relapse‐free survival

## Abstract

**Background:**

For early‐stage non‐small cell lung cancer (NSCLC), surgical resection is considered the most effective treatment strategy and curative treatment. Unfortunately, even after complete resection, almost half of all patients with stage I–IIIA NSCLC relapse and die. Although the possibility of a cure for postoperative recurrence of NSCLC is significantly low, the course of subsequent treatment can possibly affect overall survival (OS). Here, we examined the association of relapse‐free survival (RFS) and post‐progression survival (PPS) with OS in patients with postoperative recurrence of NSCLC.

**Methods:**

We evaluated 128 patients with NSCLC who underwent complete resection between January 2007 and December 2018. The association between RFS and PPS on OS was examined at the patient level.

**Results:**

Spearman's rank correlation and linear regression analyses revealed that PPS was strongly correlated with OS (*r* = 0.83, *p* < 0.05, *R* ^*2*^ = 0.72), whereas RFS was weakly associated with OS (*r* = 0.56, *p* < 0.05, *R* ^*2*^ = 0.37). Additionally, the performance status at relapse and administration of tyrosine kinase inhibitors were significantly correlated with PPS.

**Conclusions:**

PPS was significantly more strongly correlated with OS than was RFS in patients with postoperative recurrence of NSCLC. These results suggest that therapy following postoperative recurrence affects OS. Therefore, it is necessary to validate these promising results in a large prospective study.

## Graphical abstract

Post‐progression survival was strongly correlated with overall survival, whereas relapse‐free survival was weakly associated with overall survival. Additionally, the performance status at relapse and administration of tyrosine kinase inhibitors were significantly correlated with post‐progression survival. Our findings demonstrate that the course of postoperative relapse in non‐small cell lung cancer is strongly associated with post‐progression survival.

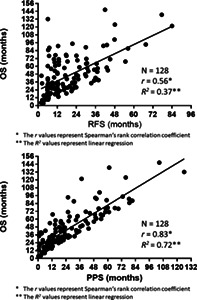

## INTRODUCTION

Lung cancer is the most common cause of cancer‐related mortalities globally, with non‐small cell lung cancer (NSCLC) accounting for approximately 80% of all lung cancers.^1^ For early‐stage NSCLC, surgical resection is considered the most effective treatment modality and curative treatment. Unfortunately, even after complete resection, almost half of all patients with stage I–IIIA NSCLC experience recurrence and die.^2^, ^3^ In fact, the median survival after postoperative recurrence of NSCLC has been reported to be between 8.1 and 17.7 months,^3^, ^4^ and the possibility of developing a cure for postoperative relapse of NSCLC is significantly low. The most promising treatment strategies for postoperative relapse of NSCLC include slowing the disease progression, providing palliative clinical care, and maintaining a patient's quality of life.

As the number of available treatment choices for NSCLC increases annually, it is possible that the effects of the early‐line treatment on overall survival (OS) may be influenced by subsequent therapies.^5^ The results from phase III trials in NSCLC have shown that prolonged progression‐free survival (PFS) does not necessarily lead to prolonged OS.^6^ This means that the PFS of front‐line treatment is not an ideal alternative measure of OS. Instead, it has been reported that post‐progression survival (PPS), which is OS minus PFS, is highly associated with OS,^7^ especially after the advent of molecular targeted agents, such as epidermal growth factor receptor (EGFR)‐tyrosine kinase inhibitors (TKIs).^8^, ^9^ We have previously reported that PPS (also known as SPP) is more strongly correlated with OS than is PFS in NSCLC.^10^, ^11^, ^12^ This suggests that subsequent therapy after disease progression has a strong effect on OS in patients with NSCLC.

According to the Japanese Lung Cancer Registry Study, postoperative relapse is the most common cause of postoperative mortality.^13^ Furthermore, most of the postoperative recurrences are distant metastases or locoregional recurrences, and the treatment of these cases is often based on the treatment strategy of locally advanced or metastatic NSCLC. Therefore, the treatments for recurrent lesions in surgically resected recurrent cases, the course of disease after recurrence, and the associated prognostic factors have not been evaluated in detail.^3^, ^14^, ^15^ However, recent advancements in imaging, appropriate postoperative adjuvant chemotherapy, high‐precision radiotherapy, and anticancer drugs, including molecular targeted therapy and immune checkpoint inhibitors (ICIs), have led to the expectation of long‐term survival in patients with NSCLC. In addition, several factors, including different patient backgrounds and treatment modalities, are thought to influence outcomes after postoperative relapse.

Taken together, this suggests that it is clinically meaningful to assess the effect of postoperative recurrence therapy on OS at the patient level. Importantly, previous studies of advanced NSCLC have reported that PPS, but not PFS, is highly associated with OS after the first‐ and second‐line treatment at the individual level.^16^ As a result, treatment after postoperative relapse may have a significant effect on OS, although the association between PPS and OS in patients with postoperative recurrence of NSCLC remains unknown. Thus, it is clinically meaningful to examine the degree to which either relapse‐free survival (RFS) or PPS is associated with OS at the individual level.

Thus, we analyzed the association between RFS and PPS on OS in patients with postoperative recurrence of NSCLC. Moreover, we also examined the prognostic influence of patient backgrounds for PPS in the postoperative recurrence of NSCLC.

## METHODS

### Patients

The study was approved by the appropriate ethics committee. All procedures complied with the ethical standards of the institutional and/or national research committee and with the 1964 Declaration of Helsinki and its later amendments or comparable ethical standards. Due to the retrospective nature of the study, the requirement for informed consent was waived by the ethics committee. However, the opportunity to refuse participation through the opt‐out method was guaranteed. This analysis included patients with postoperative recurrence of NSCLC who underwent complete resection at the Gunma Prefectural Cancer Center between January 2007 and December 2018. Each patient's pathological diagnosis was assessed based on the World Health Organization's classification. The disease stage was used according to the American Joint Committee on Cancer's tumor‐node‐metastasis (TNM) staging system.^17^ The eligibility criteria included histological evidence of NSCLC and postoperative relapse after complete resection. Moreover, only lobectomies were included, with no cases of wedge resection or segmentectomy. However, the exclusion criteria were as follows: surgical resections in other hospitals, cases of incomplete surgical resection, and incomplete medical records data. At the time of postoperative recurrence, each patient received a pretreatment physical diagnosis and underwent chest radiography, chest and abdominal computed tomography, ^18^F‐fluorodeoxyglucose positron emission tomography or bone scintigraphy, and brain magnetic resonance imaging or computed tomography for disease staging (TNM classification). Medical data for each patient were collected from their paper or electronic medical charts. In addition, treatment chemotherapeutic regimens, radiotherapy records, and subsequent‐line treatments (if administered) were also collected. Front‐ and subsequent‐line treatments were selected by the principal physician and continued until disease progression, unacceptable adverse events, or patient refusal. Following postoperative relapse, patients were allowed to select any therapies after the first‐line treatment. This investigation was approved by the Ethics Committee of the Gunma Prefectural Cancer Center. As this was a retrospective study, the ethics committee waived the need for informed consent.

### Method for evaluating treatment response

The tumor response to treatment was judged to be the best overall response. Radiographic response to treatment was assessed according to the Response Evaluation Criteria in Solid Tumors (RECIST) version 1.1^18^ as follows: complete response: disappearance of all target lesions; partial response (PR): ≥30% decrease in the sum of the target lesion diameters with the summed baseline diameters as a reference; progressive disease (PD): ≥20% increase in the sum of the target lesion diameters with the smallest sum observed during the study serving as reference or appearance of new lesion(s); and stable disease: insufficient shrinkage to qualify as PR and insufficient expansion to qualify as PD.

### Statistical analysis

RFS was measured from the surgery to the first instance of recurrence or to death for any cause. OS was measured from the surgery until death, or censoring at the last clinical examination. PPS was measured from tumor recurrence after surgery until death or until censoring at the last clinical examination. The Kaplan–Meier method was used for survival analyses. We used Spearman's rank correlation and linear regression analyses to examine whether RFS and/or PPS was correlated with OS. A Cox proportional hazards model with stepwise regression was used to detect factors that predicted PPS and to estimate hazard ratios and 95% confidence intervals. Statistical significance was set at *p* < 0.05. The two‐tailed significance level was set at *p* < 0.05. All statistical analyses were performed using JMP software for Windows (version 11.0; SAS Institute).

## RESULTS

### Patient selection and characteristics

**FIGURE 1:**
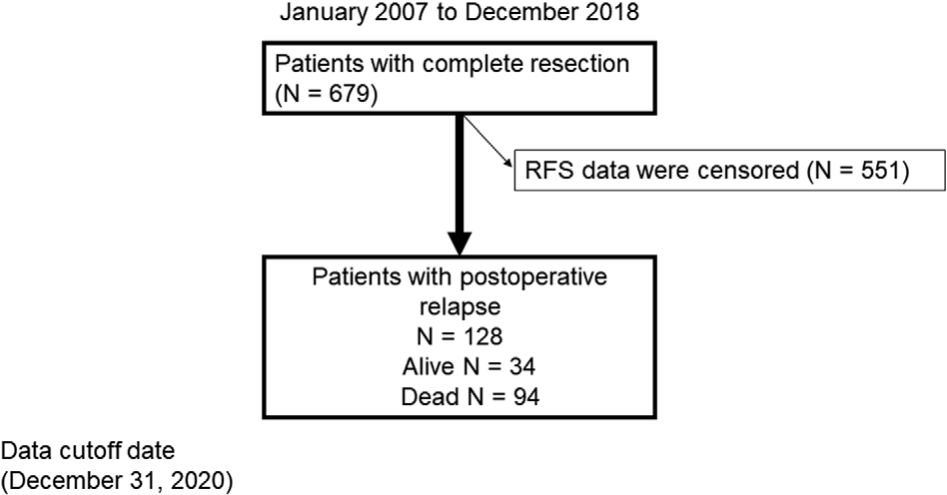
Diagram showing patient selection. The patients underwent surgical resection between January 2007 and December 2018. RFS, relapse‐free survival

From January 2007 to December 2018, 679 complete resections were performed, and as of December 31, 2020, 551 patients had no recurrence and were excluded from the analysis. Therefore, 128 patients with postoperative relapse were included in the analysis (Figure 1). Of the 128 patients in the analysis cohort, 94 died and 34 were alive at the data cutoff. The median follow‐up duration was 37.0 (range, 3.5–146.5) months. The median age at relapse was 71 (range, 41–81) years. Baseline patient characteristics are summarized in Table 1. The patient cohort (median age at relapse, 71 [range, 41–88] years) was predominately male. Of the 128 patients, 112 and 16 had performance status (PS) 0–1 and 2–3, respectively. Postoperative adjuvant chemotherapy was administered to 47 of the 128 patients, and the breakdown of postoperative adjuvant chemotherapy is shown in Table **S1**.

**TABLE 1:** Baseline patient characteristics at postoperative relapse

Characteristic	*N* = 128
Sex	
Male/female	93/35
Median age at operation (years)	69 (41–85)
Median age at relapse (years)	71 (41–88)
Performance status	
0/1/2/3/4	62/50/10/6/0
Smoking history	
Yes/no	96/32
Histology	
Adenocarcinoma/squamous cell carcinoma/others	86/28/14
Pathological stage at diagnosis	
I/II/III/IV	51/31/46/0
Operation	
Lobectomy/pneumonectomy	124/4
Driver mutation/translocation	
*EGFR*/*ALK*/*ROS‐1*/*BRAF*/others/negative or unknown	32/0/2/0/3/91
PD‐L1 TPS	
<1%/1%–49%/≥50%/unknown	18/11/7/92
Administration of adjuvant chemotherapy	
Yes/no	47/81
Administration of TKIs	
Yes/no	31/97
Administration of ICIs	
Yes/no	18/110
Recurrent pattern	
Local recurrence/distant metastasis	29/99
Intracranial metastases at recurrence	
Yes/no	23/105
Liver metastases at recurrence	
Yes/no	9/119
Bone metastases at recurrence	
Yes/no	28/100
Radiotherapy for postoperative lymph node recurrence	
Yes/no	22/106
Postoperative radiotherapy after recurrence (any site)	
Yes/no	67/61
Number of therapies after postoperative relapse	
0/1/2/3/≥4	51/34/23/13/7
Median (range)	1 (0–7)

Abbreviations: *ALK*, anaplastic lymphoma kinase; *BRAF*, v‐raf murine sarcoma viral oncogene homolog B1; *EGFR*, epidermal growth factor receptor; ICI, immune checkpoint inhibitor; *ROS‐1*, c‐ros oncogene 1; TKI, tyrosine kinase inhibitor; TPS, tumor proportion score.

### Subsequent treatment beyond postoperative relapse and treatment efficacy

**FIGURE 2:**
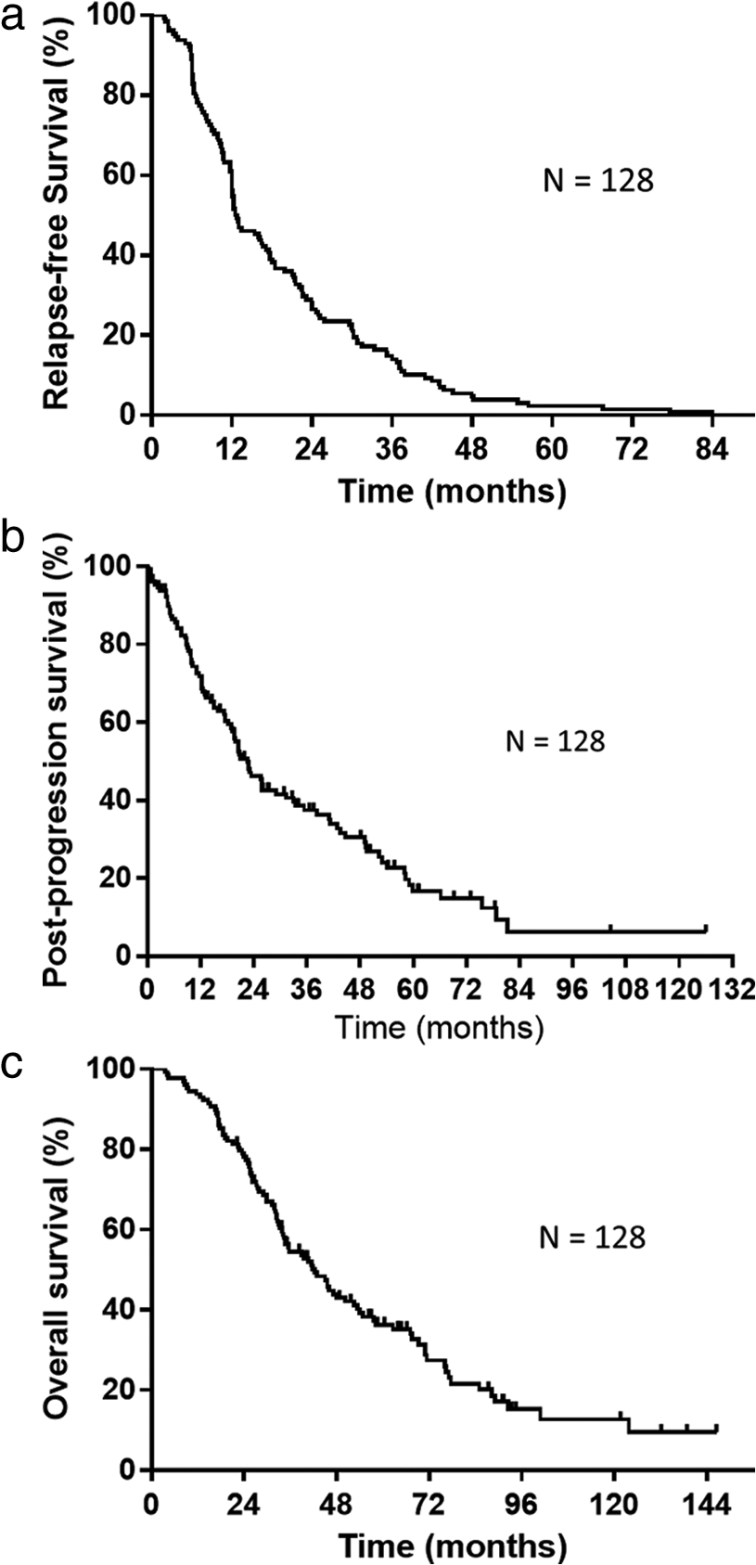
(a) Kaplan–Meier plot showing relapse‐free survival (RFS). Median RFS: 12.6 months. (b) Kaplan–Meier plots showing post‐progression survival (PPS). Median PPS: 22.5 months. (c) Kaplan–Meier plot showing overall survival (OS). Median OS: 42.2 months

The median number of therapeutic regimens beyond postoperative recurrence for the 128 patients was 1 (range, 0–7). All treatments received after postoperative recurrence are shown in Table 2. Of the 128 patients with postoperative relapse, 77 (excluding 51 who received only supportive care) received anticancer agents alone or combined with radiotherapy. Twenty‐two patients received radiotherapy for postoperative lymph node recurrence. As a first‐line treatment following postoperative recurrence, 30 patients received platinum combination chemotherapy (including two patients who received a combination of ICIs), 10 patients received docetaxel monotherapy, and 23 patients received EGFR‐TKI. Of the 34 driver mutation/translocation positive cases (32 *EGFR* mutation and 2 ROS‐1 translocation), 29 were treated with TKIs during the course of postoperative recurrence. The median RFS, PPS, and OS were 12.6, 22.5, and 42.2 months, respectively (Figure 2(a)–(c)).

**TABLE 2:** Number of chemotherapy regimens used as subsequent treatment beyond postoperative relapse

	First‐line	Second‐line	Third‐line	≥Fourth‐line	Total
Platinum combination	28	8	3	1	40
Platinum combination + ICIs	2	1	0	0	3
Docetaxel	10	8	1	0	19
Docetaxel + ramucirumab	1	1	2	0	4
Pemetrexed	1	7	3	0	11
S1	0	0	2	4	6
Vinorelbine	0	1	0	2	3
Gemcitabine	1	0	0	2	3
Amurubicin	1	4	0	2	7
First‐ or second‐generation EGFR‐TKIs	23	3	0	0	26
First‐ or second‐generation EGFR‐TKI rechallenge	‐	3	0	0	3
Osimertinib	3	1	2	0	6
Other tyrosine kinase inhibitors	2	0	0	0	2
Immune check point monotherapy	2	7	5	1	15
Chemoradiotherapy	2	0	0	0	2
Others (single agents)	1	0	2	1	4
Investigational agents	0	0	1	0	1
Best supportive care	51	‐	‐	‐	51

Abbreviations: EGFR‐TKIs, epidermal growth factor receptor‐tyrosine kinase inhibitors; ICI, immune checkpoint inhibitor; TKI, tyrosine kinase inhibitor.

### Associations between relapse‐free survival and post‐progression survival (PPS) and overall survival

**FIGURE 3:**
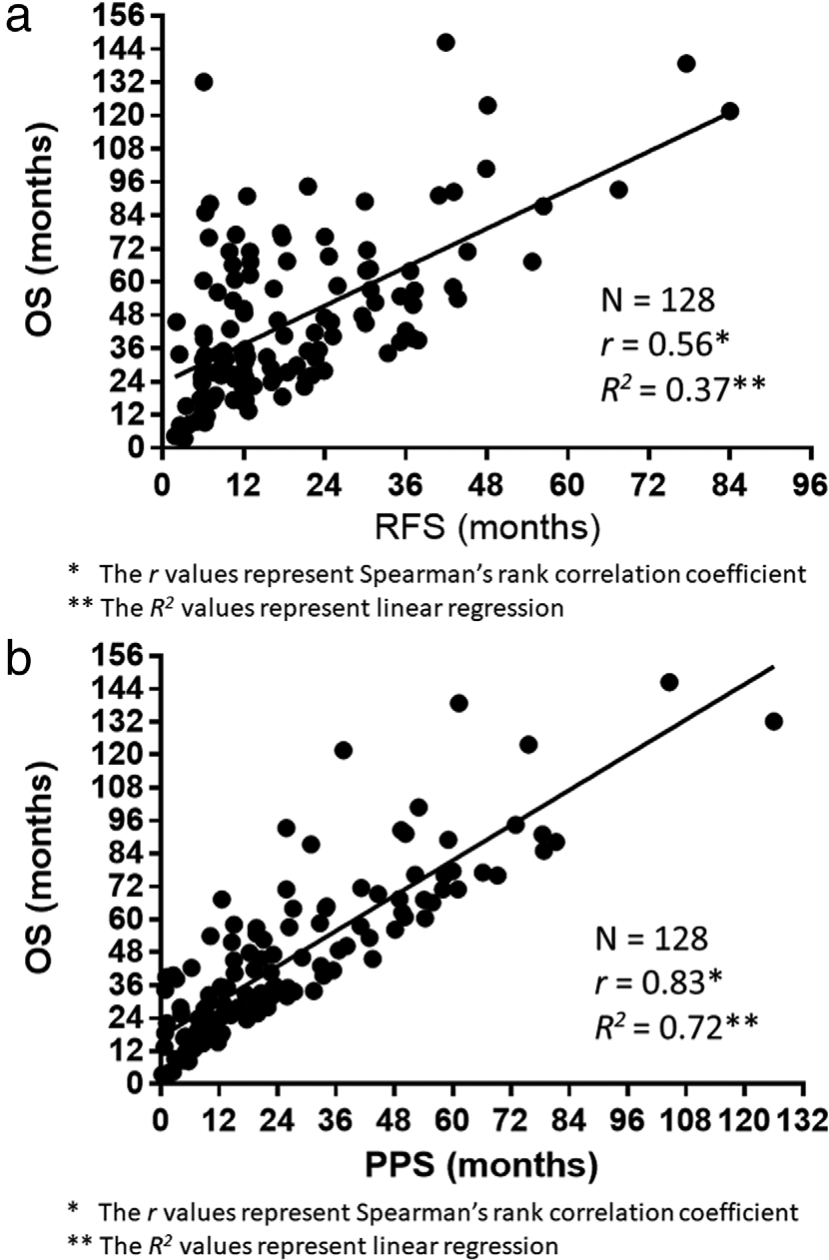
(a) Correlation between the overall survival (OS) and relapse‐free survival. (b) Correlation between the OS and post‐progression survival

The associations between RFS and PPS and OS are demonstrated in Figure 3(a),(b). Spearman's rank correlation coefficients and linear regression analyses demonstrated that PPS was strongly correlated with OS (*r* = 0.83, *p* < 0.0001, *R* ^*2*^ = 0.72), whereas RFS was moderately correlated with OS (*r* = 0.56, *p* < 0.0001, *R* ^*2*^ = 0.37).

### Factors affecting PPS

**FIGURE 4:**
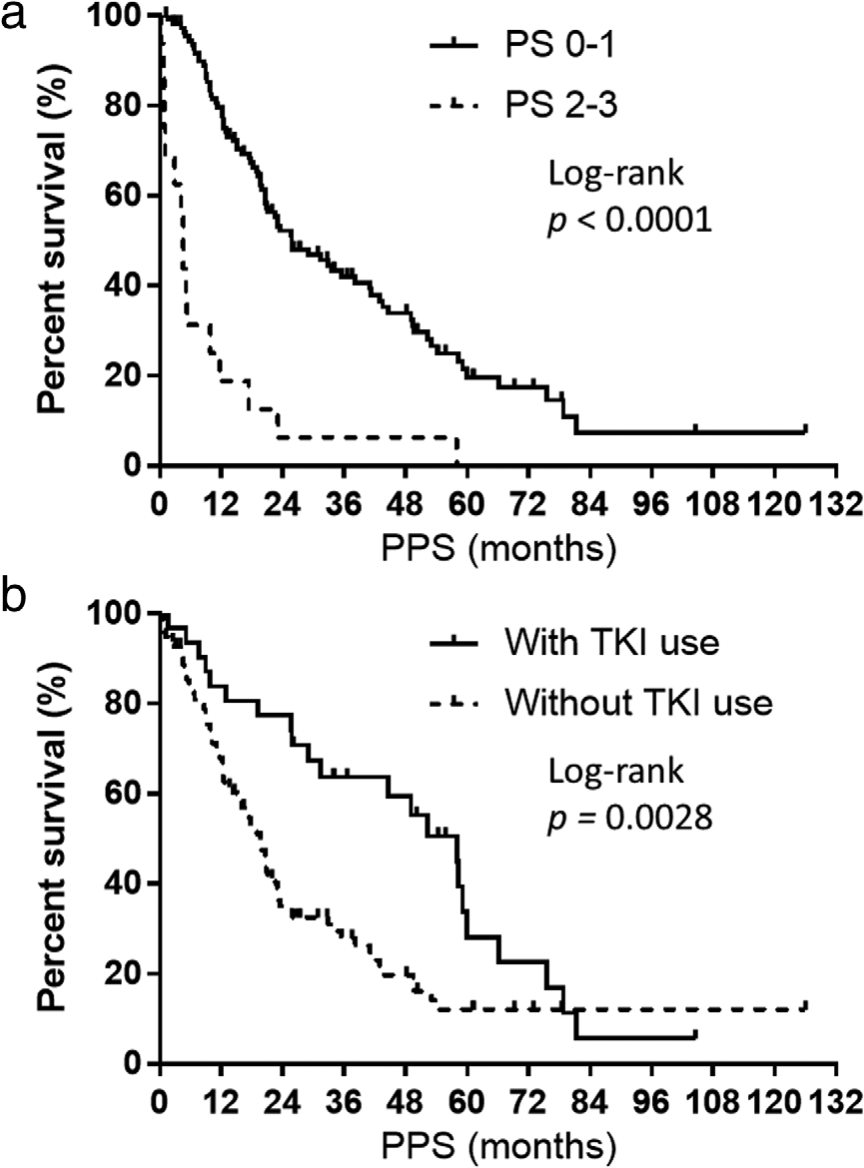
(a) Kaplan–Meier plots showing post‐progression survival (PPS) according to performance status (PS) at relapse. PS 0–1, median = 25.8 months; PS 2–3, median = 4.5 months. (b) Kaplan–Meier plots showing PPS according to tyrosine kinase inhibitor (TKI) administration. With TKI administration, median = 57.9 months; without TKI administration, median = 19.5 months

Given that the PPS was associated with OS, the association between PPS and different factors was analyzed. Univariate analysis showed that PS at relapse, administration of adjuvant chemotherapy, and administration of TKI were significantly associated with PPS (*p* < 0.05; Table 3). In addition, the multivariate analysis of these factors revealed that PS at relapse and administration of TKI were independently correlated with PPS (*p* < 0.05; Table 3). Log‐rank tests indicated that PPS differed among patients according to PS at relapse and TKI administration. According to PS at relapse, the PPS in patients with a PS of 0–1 and 2–3 were 25.8 and 4.5 months, respectively (log‐rank tests, *p* < 0.0001; Figure 4(a)). Regarding administration of TKIs, the PPS in patients with and without TKIs were 57.9 and 19.5 months, respectively (log‐rank tests, *p* = 0.0028; Figure 4(b)). For further analysis, we performed univariate and multivariate analyses by excluding patients who received TKIs and ICIs. The univariate and multivariate analyses of the 97 patients who were not treated with TKIs are shown in Table S2, the univariate and multivariate analyses of the 31 patients who were treated with TKIs are shown in Table S3, and the univariate and multivariate analyses of the 110 patients who were not treated with ICIs are shown in Table S4. Moreover, the univariate and multivariate analyses of the 47 patients who were treated with postoperative adjuvant chemotherapy are shown in Table S5, the univariate and multivariate analyses of the 81 patients who were not treated with postoperative adjuvant chemotherapy are shown in Table S6, and the univariate and multivariate analyses of the 97 patients who were PS 0–1 at relapse are shown in Table S7. Multivariate analysis revealed that PS at relapse was an independent prognostic factor in the cohort, excluding those who received TKIs (*p* < 0.05; Table S2). The only significant difference indicated by univariate analysis was the presence of bone metastases in the cohort, including those who received TKIs (*p* < 0.05; Table S3). Multivariate analysis revealed that PS at relapse, administration of TKI, and the presence of bone metastases at recurrence were independent prognostic factors in the cohort, excluding patients who received ICIs (*p* < 0.05; Table S4). Multivariate analysis revealed that PS at relapse and the presence of liver metastases at recurrence were independent prognostic factors in the cohort, including patients who were treated with postoperative adjuvant chemotherapy (*p* < 0.05; Table S5). Multivariate analysis revealed that PS at relapse was an independent prognostic factor in the cohort, excluding patients who were treated with postoperative adjuvant chemotherapy (*p* < 0.05; Table S6). Multivariate analysis revealed that administration of TKI was an independent prognostic factor in the cohort who were PS 0–1 at relapse (*p* < 0.05; Table S7).

**TABLE 3:** Univariate and multivariate Cox regression analyses of patient baseline characteristics

Factors	Post‐progression survival					
	Univariate analysis			Multivariate analysis		
	HR	95% CI	*p*‐value	HR	95% CI	*p*‐value
Sex						
Male/female	1.41	0.90–2.32	0.13			
Pathological stage at diagnosis						
I/II–III	0.92	0.60–1.41	0.73			
Age at relapse (years)						
<75/≥75	0.97	0.62–1.55	0.9			
PS at relapse						
0–1/≥2	0.2	0.12–0.37	**<0.0001**	0.18	0.10–0.33	**<0.0001**
Smoking history						
Yes/no	1.46	0.90–2.50	0.12			
Histology						
Adenocarcinoma/nonadenocarcinoma	0.65	0.42–1.00	0.05			
Administration of adjuvant chemotherapy						
Yes/no	0.55	0.35–0.84	**0.006**	0.65	0.40–1.03	0.07
Administration of platinum combination chemotherapy						
Yes/no	0.97	0.63–1.48	0.9			
Administration of TKI						
Yes/no	0.47	0.28–0.76	**0.002**	0.51	0.29–0.85	**0.009**
Administration of ICIs						
Yes/no	0.66	0.33–1.19	0.18			
Recurrent pattern						
Local recurrence/distant metastasis	0.69	0.41–1.10	0.12			
Intracranial metastases at recurrence						
Yes/no	0.79	0.44–1.32	0.38			
Liver metastases at recurrence						
Yes/no	1.98	0.87–3.88	0.09			
Bone metastases at recurrence						
Yes/no	1.56	0.95–2.48	0.07			
Radiotherapy for postoperative lymph node recurrence						
Yes/no	0.59	0.32–1.00	0.05			

*Note*: *p*‐values in bold are considered statistically significant (*p* < 0.05).

Abbreviations: CI, confidence interval; HR, hazard ratio; ICI, immune checkpoint inhibitor; PS, performance status; TKI, tyrosine kinase inhibitor.

## DISCUSSION

In the current analysis, we investigated the association of individual‐level RFS and PPS with OS in patients with postoperative recurrence of NSCLC. We found that PS at relapse and TKI administration were significant independent prognostic factors for PPS. Importantly, many of the patients with postoperative relapse had undergone regular follow‐up after surgical resection, which may have resulted in lower tumor volume compared with patients with NSCLC with metastases already present at the time of diagnosis. This difference or heterogeneity in tumor volume may have a favorable effect on the PFS and OS of front‐line treatment after recurrence in patients with postoperative NSCLC relapse.^19^

Endpoints from various clinical trials were assessed in previous meta‐analyses,^20^, ^21^ and biostatisticians have proposed several metrics to identify alternative endpoints.^22^, ^23^ A report has found that PPS, defined as survival after disease progression, was important in assessing the validity of OS as a trial endpoint.^7^ PFS is usually defined as the survival period without “progression or death for any reason,” whereas RFS is usually defined as the survival period without “relapse or death for any reason” in the cancer‐free state beyond operation. Previous reports in patients with NSCLC have shown that PPS is strongly associated with OS after first‐, second‐, and third‐line treatment.^8^, ^9^, ^24^ Furthermore, our previous reports have shown that individual‐level data on PPS are appropriate for assessing OS in patients with advanced NSCLC after front‐line treatment (first‐ and second‐line).^10^, ^11^, ^12^ Therefore, we analyzed the correlation of RFS and PPS with OS in patients with postoperative recurrence of NSCLC. Our findings suggest that RFS does not associate strongly with OS and that prolonged RFS does not necessarily result in prolonged OS. Furthermore, RFS was considerably shorter than PPS in this analysis cohort. In fact, we found that PPS was strongly associated with OS, suggesting that future trials should consider the clinical factors that may affect PPS.

Several studies have shown that PPS is strongly associated with OS in front‐line treatment and that the factors affecting PPS include PS and response to anticancer drugs.^10^, ^11^, ^12^ However, the clinical factors that influence PPS at the individual patient level in the postoperative recurrence of NSCLC remain poorly understood. In the present study, multivariate analysis demonstrated that PS at relapse and TKI administration were strongly associated with PPS. The results of this analysis suggest that PS at relapse is important for prolonging PPS in patients with postoperative recurrence of NSCLC. The large number of anticancer drugs administered after postoperative relapse may be attributed to the large number of treatment options for first‐ and subsequent‐line treatments of NSCLC, including platinum‐based combination regimens, cytotoxic drug monotherapy, ICIs, and EGFR‐TKIs (gefitinib, erlotinib, afatinib, and osimertinib) (Table 2). In the present multivariate analysis of the overall population, the administration of TKIs was an independent prognostic factor. Importantly, patients with postoperative relapse harboring sensitive *EGFR* mutations have been reported to have a better prognosis than those without sensitive *EGFR* mutations.^25^ In our study, many patients died before examination for the T790M mutation, but if many patients with secondary T790M *EGFR* mutations are administered with osimertinib, the effect on PPS may be higher than expected. Given that the front‐ and subsequent‐line treatments are changing, the PPS of these patients after postoperative relapse may also change. For example, when osimertinib is treated as a second‐line treatment in addition to first‐line treatment with first‐ or second‐generation EGFR‐TKIs, survival after progression has a stronger effect on the OS of patients with secondary T790M mutation‐positive NSCLC; however, PPS may also be of value when osimertinib is used as a first‐line treatment beyond postoperative recurrence. Previous reports have suggested that OS correlates better with PPS than RFS in patients harboring *EGFR*‐positive NSCLC who have undergone complete resection.^26^ There is a report of significant disease‐free survival (DFS) prolongation with the administration of osimertinib as postoperative adjuvant chemotherapy in *EGFR*‐mutated NSCLC.^27^ However, if PPS is shown to alter OS in patients with postoperative recurrence, it is possible that DFS prolongation with adjuvant EGFR‐TKIs does not affect OS. At this time, the use of EGFR‐TKIs as postoperative adjuvant chemotherapy may remain controversial. Additionally, in the current univariate analysis, radiotherapy for postoperative lymph node recurrence tended to have a better prognosis for PPS, although this was not statistically significant. This may also be due to the small cohort size. Furthermore, when TKI‐treated patients, who were mainly driver gene mutation/translocation positive, were excluded from the analysis, the PS at postoperative relapse was also an independent prognostic factor (Table S2). In fact, when ICI‐treated patients were excluded from the analysis, PS at postoperative relapse, administration of TKI, and presence of bone metastases were independent prognostic factors (Table S4). Moreover, in patients who were treated with postoperative adjuvant chemotherapy, PS at postoperative relapse and presence of liver metastases were independent prognostic factors (Table S5), and in patients who were not treated with postoperative adjuvant chemotherapy, PS at postoperative relapse was the independent prognostic factor (Table S6). Overall, PS at postoperative relapse was the only prognostic factor for PPS in multivariate analysis. Therefore, a favorable postoperative PS at relapse suggests a favorable subsequent disease course. Furthermore, administration of TKI was an independent prognostic factor only in patients with a good PS of 0–1 at relapse. Thus, PS at relapse and administration of an EGFR‐TKI are consistently important prognostic factors in the various subgroups. Figure 4(a),(b) indicate that in the overall population, PPS differed among patients according to PS at relapse and TKI administration, respectively. PS has been reported to be a powerful prognostic factor,^28^, ^29^ but even for patients with postoperative relapse, such as the present cohort, PS at relapse has a significant effect on the subsequent course of the disease. Regarding TKI administration being an independent prognostic factor, this may be due to the fact that TKIs were administered in more than 85% of the driver gene mutation/translocation positive cases (most of which were sensitive *EGFR* mutation positive) in this cohort. This is because *EGFR* mutations are the strongest predictive biomarker for PFS and tumor response to EGFR‐TKI treatment.^30^

Importantly, the presence or absence of postoperative adjuvant chemotherapy was consistently statistically significant for PPS in univariate analysis, but not in multivariate analysis, whether in the overall population, excluding patients receiving TKIs, or excluding patients receiving ICI. Although the reason for this remains unclear, this could be attributable to the small cohort size.

The present study has several limitations. First, although the current analysis included cases of postoperative relapse after 2007, not all driver mutation/translocations were identified. Many cases of *ALK*, *ROS‐1*, and *BRAF* mutation/translocation‐positive NSCLC have not been tested in clinical practice, and it is possible that some of the unknown cases may have driver mutation/translocation. Therefore, this study should be interpreted with this in mind. Second, there is a lack of uniformity in the treatment of postoperative relapse, including anticancer drug therapy and radiotherapy. Nevertheless, we believe that the current analysis, which is based on real practice, is clinically meaningful. Third, the date of disease progression was assessed by the treating physician, which may have led to variability in RFS. However, this variability is an unavoidable feature of retrospective studies. Fourth, although we included censored data on survival without a death event, we are confident that this did not affect the results as the RFS remained unchanged even in patients who did not die. Moreover, if PPS and OS are further prolonged, the correlation between PPS and OS will become even stronger.

In conclusion, PPS is more strongly correlated with OS than is RFS in patients with postoperative recurrence of NSCLC. PS at relapse and TKI administration were also significantly correlated with PPS. These results demonstrate that the clinical course of postoperative recurrence in NSCLC is strongly associated with PPS. Nevertheless, future large cohort prospective trials are needed to validate the present results in other patient cohorts.

## CONFLICT OF INTEREST

The authors declare no conflicts of interest.

## Supporting information


**Table S1.** Supporting information
