# Supplementary material for: Course of postoperative relapse in non‐small cell lung cancer is strongly associated with post‐progression survival
Source: Thorac Cancer. 2021 Sep 3;12(20):2740–8. doi: 10.1111/1759-7714.14119 (PMC8520813; doi:10.1111/1759-7714.14119)
Supplement: Supplementary file 1 — Table S1. Supporting information [file TCA-12-2740-s001.docx]

**Supplementary Table 1.** Chemotherapeutic regimens used during postoperative adjuvant chemotherapy

| Adjuvant chemotherapy | N |
| --- | --- |
| Tegafur/uracil | 12 |
| Cisplatin plus vinorelbine | 28 |
| Others | 7 |
| None | 81 |

**Supplementary Table 2.** Univariate and multivariate Cox regression analyses of 97 patients who were not treated with TKIs

|  | Survival post-progression | | | | | |
| --- | --- | --- | --- | --- | --- | --- |
|  | Univariate analysis | | | Multivariate analysis | | |
| Factors | HR | 95% CI | *P*-value | HR | 95% CI | *P*-value |
| Sex |  |  |  |  |  |  |
| Male/female | 1.58 | 0.88–3.10 | 0.12 |  |  |  |
| Pathological stage at diagnosis |  |  |  |  |  |  |
| I/II–III | 0.88 | 0.54–1.40 | 0.59 |  |  |  |
| Age at relapse (years) |  |  |  |  |  |  |
| <75/≥75 | 1.12 | 0.68–1.93 | 0.65 |  |  |  |
| PS at relapse |  |  |  |  |  |  |
| 0–1/≥2 | 0.15 | 0.08–0.30 | **<0.0001** | 0.18 | 0.10–0.37 | **<0.0001** |
| Smoking history |  |  |  |  |  |  |
| Yes/no | 1.22 | 0.65–2.55 | 0.53 |  |  |  |
| Histology |  |  |  |  |  |  |
| Adenocarcinoma/non-adenocarcinoma | 0.81 | 0.51–1.30 | 0.4 |  |  |  |
| Administration of adjuvant chemotherapy |  |  |  |  |  |  |
| Yes/no | 0.58 | 0.32–0.98 | **0.04** | 0.73 | 0.41–1.27 | 0.28 |
| Administration of platinum combination chemotherapy |  |  |  |  |  |  |
| Yes/no | 1.09 | 0.65–1.78 | 0.72 |  |  |  |
| Administration of ICIs |  |  |  |  |  |  |
| Yes/no | 0.47 | 0.22–0.91 | **0.02** | 0.59 | 0.27–1.15 | 0.13 |
| Recurrent pattern |  |  |  |  |  |  |
| Local recurrence/distant metastasis | 0.67 | 0.37–1.13 | 0.14 |  |  |  |
| Intracranial metastases at recurrence |  |  |  |  |  |  |
| Yes/no | 0.73 | 0.36–1.35 | 0.34 |  |  |  |
| Liver metastases at recurrence |  |  |  |  |  |  |
| Yes/no | 1.82 | 0.63–4.13 | 0.23 |  |  |  |
| Bone metastases at recurrence |  |  |  |  |  |  |
| Yes/no | 1.65 | 0.86–2.93 | 0.12 |  |  |  |
| Radiotherapy for postoperative lymph node recurrence |  |  |  |  |  |  |
| Yes/no | 0.61 | 0.30–1.13 | 0.12 |  |  |  |

HR, hazard ratio; CI, confidence interval; PS, performance status; ICI, immune checkpoint inhibitor; TKI, tyrosine kinase inhibitor

*P*-values in bold are considered statistically significant (*p* < 0.05).

**Supplementary Table 3.** Univariate and multivariate Cox regression analyses of 31 patients who were treated with TKIs

|  | Survival post-progression | | | | | |
| --- | --- | --- | --- | --- | --- | --- |
|  | Univariate analysis | | | Multivariate analysis | | |
| Factors | HR | 95% CI | *P*-value | HR | 95% CI | *P*-value |
| Sex |  |  |  |  |  |  |
| Male/female | 0.75 | 0.56–3.12 | 0.51 |  |  |  |
| Pathological stage at diagnosis |  |  |  |  |  |  |
| I/II–III | 1.35 | 0.45–3.83 | 0.56 |  |  |  |
| Age at relapse (years) |  |  |  |  |  |  |
| <75/≥75 | 0.47 | 0.17–1.35 | 0.15 |  |  |  |
| PS at relapse |  |  |  |  |  |  |
| 0–1/≥2 | 0.28 | 0.08–1.26 | 0.09 |  |  |  |
| Smoking history |  |  |  |  |  |  |
| Yes/no | 1.05 | 0.45–2.58 | 0.90 |  |  |  |
| Administration of adjuvant chemotherapy |  |  |  |  |  |  |
| Yes/no | 0.42 | 0.14–1.18 | 0.09 |  |  |  |
| Administration of platinum combination chemotherapy |  |  |  |  |  |  |
| Yes/no | 0.51 | 0.18–1.27 | 0.15 |  |  |  |
| Administration of ICIs |  |  |  |  |  |  |
| Yes/no | 2.01 | 0.30–7.67 | 0.40 |  |  |  |
| Recurrent pattern |  |  |  |  |  |  |
| Local recurrence/distant metastasis | 0.40 | 0.11–1.17 | 0.10 |  |  |  |
| Intracranial metastases at recurrence |  |  |  |  |  |  |
| Yes/no | 1.35 | 0.43–3.60 | 0.57 |  |  |  |
| Liver metastases at recurrence |  |  |  |  |  |  |
| Yes/no | 4.50 | 0.94–17.26 | 0.05 |  |  |  |
| Bone metastases at recurrence |  |  |  |  |  |  |
| Yes/no | 3.74 | 1.45–9.99 | **0.006** |  |  |  |
| Radiotherapy for postoperative lymph node recurrence |  |  |  |  |  |  |
| Yes/no | 0.40 | 0.11–1.17 | 0.10 |  |  |  |

HR, hazard ratio; CI, confidence interval; PS, performance status; TKI, tyrosine kinase inhibitor; ICI, immune checkpoint inhibitor

*P*-values in bold are considered statistically significant (*p* < 0.05).

**Supplementary Table 4.** Univariate and multivariate Cox regression analyses of 110 patients who were not treated with immune checkpoint inhibitors

|  | Survival post-progression | | | | | |
| --- | --- | --- | --- | --- | --- | --- |
|  | Univariate analysis | | | Multivariate analysis | | |
| Factors | HR | 95% CI | *P*-value | HR | 95% CI | *P*-value |
| Sex |  |  |  |  |  |  |
| Male/female | 1.36 | 0.84–2.32 | 0.21 |  |  |  |
| Pathological stage at diagnosis |  |  |  |  |  |  |
| I/II –III | 0.99 | 0.62–1.55 | 0.97 |  |  |  |
| Age at relapse (years) |  |  |  |  |  |  |
| <75/≥75 | 0.85 | 0.53–1.38 | 0.51 |  |  |  |
| PS at relapse |  |  |  |  |  |  |
| 0–1/≥2 | 0.22 | 0.13–0.40 | **<0.0001** | 0.24 | 0.13–0.44 | **<0.0001** |
| Smoking history |  |  |  |  |  |  |
| Yes/No | 1.41 | 0.84–2.49 | 0.18 |  |  |  |
| Histology |  |  |  |  |  |  |
| Adenocarcinoma/non-adenocarcinoma | 0.68 | 0.43–1.07 | 0.1 |  |  |  |
| Administration of adjuvant chemotherapy |  |  |  |  |  |  |
| Yes/no | 0.51 | 0.30–0.82 | **0.005** | 0.6 | 0.35–1.01 | 0.05 |
| Administration of platinum combination chemotherapy |  |  |  |  |  |  |
| Yes/no | 0.9 | 0.54–1.43 | 0.66 |  |  |  |
| Administration of TKI |  |  |  |  |  |  |
| Yes/no | 0.41 | 0.24–0.68 | **0.0005** | 0.37 | 0.20–0.66 | **0.0006** |
| Recurrent pattern |  |  |  |  |  |  |
| Local recurrence/distant metastasis | 0.62 | 0.35–1.01 | 0.06 |  |  |  |
| Intracranial metastases at recurrence |  |  |  |  |  |  |
| Yes/no | 0.73 | 0.39–1.27 | 0.28 |  |  |  |
| Liver metastases at recurrence |  |  |  |  |  |  |
| Yes/no | 1.66 | 0.69–3.38 | 0.23 |  |  |  |
| Bone metastases at recurrence |  |  |  |  |  |  |
| Yes/no | 1.86 | 1.10–3.03 | **0.02** | 2.08 | 1.16–3.59 | **0.013** |
| Radiotherapy for postoperative lymph node recurrence |  |  |  |  |  |  |
| Yes/no | 0.53 | 0.28–0.93 | **0.02** | 0.73 | 0.38–1.33 | 0.32 |

HR, hazard ratio; CI, confidence interval; PS, performance status; TKI, tyrosine kinase inhibitor

*P*-values in bold are considered statistically significant (*p* < 0.05).

**Supplementary Table 5.** Univariate and multivariate Cox regression analyses of 47 patients who were treated with postoperative adjuvant chemotherapy

|  | Survival post-progression | | | | | |
| --- | --- | --- | --- | --- | --- | --- |
|  | Univariate analysis | | | Multivariate analysis | | |
| Factors | HR | 95% CI | *P*-value | HR | 95% CI | *P*-value |
| Sex |  |  |  |  |  |  |
| Male/female | 1.36 | 0.65–3.06 | 0.41 |  |  |  |
| Pathological stage at diagnosis |  |  |  |  |  |  |
| I/II–III | 1.19 | 0.46–2.79 | 0.69 |  |  |  |
| Age at relapse |  |  |  |  |  |  |
| <75/≥75 | 1.18 | 0.34–7.44 | 0.81 |  |  |  |
| PS at relapse |  |  |  |  |  |  |
| 0–1/≥2 | 0.19 | 0.07–0.60 | **0.007** | 0.24 | 0.07–0.87 | **0.03** |
| Smoking history |  |  |  |  |  |  |
| Yes/no | 1.55 | 0.69–3.39 | 0.29 |  |  |  |
| Histology |  |  |  |  |  |  |
| Adenocarcinoma/non-adenocarcinoma | 0.56 | 0.26–1.30 | 0.17 |  |  |  |
| Administration of platinum combination chemotherapy |  |  |  |  |  |  |
| Yes/no | 1.14 | 0.55–2.38 | 0.72 |  |  |  |
| Administration of TKI |  |  |  |  |  |  |
| Yes/no | 0.5 | 0.21–1.13 | 0.09 |  |  |  |
| Administration of ICIs |  |  |  |  |  |  |
| Yes/no | 1.07 | 0.38–2.60 | 0.88 |  |  |  |
| Recurrent pattern |  |  |  |  |  |  |
| Local recurrence/distant metastasis | 0.55 | 0.19–1.37 | 0.21 |  |  |  |
| Intracranial metastases at recurrence |  |  |  |  |  |  |
| Yes/no | 1.23 | 0.48–2.83 | 0.63 |  |  |  |
| Liver metastases at recurrence |  |  |  |  |  |  |
| Yes/no | 8.46 | 1.23–37.2 | **0.03** | 9.3 | 1.32–42.6 | **0.02** |
| Bone metastases at recurrence |  |  |  |  |  |  |
| Yes/no | 2.48 | 1.08–5.43 | **0.03** | 1.35 | 0.49–3.38 | 0.53 |
| Radiotherapy for postoperative lymph node recurrence |  |  |  |  |  |  |
| Yes/no | 0.33 | 0.09–0.93 | **0.03** | 0.41 | 0.10–1.24 | 0.12 |

HR, hazard ratio; CI, confidence interval; PS, performance status; TKI, tyrosine kinase inhibitor; ICI, immune checkpoint inhibitor

*P*-values in bold are considered statistically significant (*p* < 0.05).

**Supplementary Table 6.** Univariate and multivariate Cox regression analyses of 81 patients who were not treated with postoperative adjuvant chemotherapy

|  | Survival post-progression | | | | | |
| --- | --- | --- | --- | --- | --- | --- |
|  | Univariate analysis | | | Multivariate analysis | | |
| Factors | HR | 95% CI | *P*-value | HR | 95% CI | *P*-value |
| Sex |  |  |  |  |  |  |
| Male/female | 1.22 | 0.42–1.45 | 0.5 |  |  |  |
| Pathological stage at diagnosis |  |  |  |  |  |  |
| I/II–III | 0.72 | 0.43–1.18 | 0.19 |  |  |  |
| Age at relapse |  |  |  |  |  |  |
| <75/≥75 | 1.3 | 0.79–2.19 | 0.29 |  |  |  |
| PS at relapse |  |  |  |  |  |  |
| 0–1/≥2 | 0.17 | 0.09–0.37 | **<0.0001** | 0.2 | 0.10–0.42 | **<0.0001** |
| Smoking history |  |  |  |  |  |  |
| Yes/no | 1.29 | 0.71–2.56 | 0.4 |  |  |  |
| Histology |  |  |  |  |  |  |
| Adenocarcinoma/non-adenocarcinoma | 0.65 | 0.39–1.11 | 0.11 |  |  |  |
| Administration of platinum combination chemotherapy |  |  |  |  |  |  |
| Yes/no | 1.1 | 0.61–1.89 | 0.73 |  |  |  |
| Administration of TKI |  |  |  |  |  |  |
| Yes/no | 0.47 | 0.22–0.92 | **0.02** | 0.55 | 0.25–1.09 | 0.09 |
| Administration of ICIs |  |  |  |  |  |  |
| Yes/no | 0.53 | 0.18–1.21 | 0.14 |  |  |  |
| Recurrent pattern |  |  |  |  |  |  |
| Local recurrence/distant metastasis | 0.63 | 0.34–1.10 | 0.1 |  |  |  |
| Intracranial metastases at recurrence |  |  |  |  |  |  |
| Yes/no | 0.69 | 0.31–1.34 | 0.29 |  |  |  |
| Liver metastases at recurrence |  |  |  |  |  |  |
| Yes/no | 1.3 | 0.50–2.81 | 0.55 |  |  |  |
| Bone metastases at recurrence |  |  |  |  |  |  |
| Yes/no | 1.37 | 0.71–2.45 | 0.32 |  |  |  |
| Radiotherapy for postoperative lymph node recurrence |  |  |  |  |  |  |
| Yes/no | 0.65 | 0.82–3.08 | 0.18 |  |  |  |

HR, hazard ratio; CI, confidence interval; PS, performance status; TKI, tyrosine kinase inhibitor; ICI, immune checkpoint inhibitor

*P*-values in bold are considered statistically significant (*p* < 0.05).

**Supplementary Table 7.** Univariate and multivariate Cox regression analyses of 97 patients who were PS 0–1 at relapse

|  | Survival post-progression | | | | | |
| --- | --- | --- | --- | --- | --- | --- |
|  | Univariate analysis | | | Multivariate analysis | | |
| Factors | HR | 95% CI | *P*-value | HR | 95% CI | *P*-value |
| Sex |  |  |  |  |  |  |
| Male/female | 1.51 | 0.90–2.68 | 0.11 |  |  |  |
| Pathological stage at diagnosis |  |  |  |  |  |  |
| I/II–III | 0.97 | 0.60–1.55 | 0.92 |  |  |  |
| Age at relapse |  |  |  |  |  |  |
| <75/≥75 | 0.88 | 0.54–1.45 | 0.61 |  |  |  |
| Smoking history |  |  |  |  |  |  |
| Yes/no | 1.54 | 0.89–2.86 | 0.12 |  |  |  |
| Histology |  |  |  |  |  |  |
| Adenocarcinoma/non-adenocarcinoma | 0.62 | 0.39–1.00 | 0.05 |  |  |  |
| Administration of adjuvant chemotherapy |  |  |  |  |  |  |
| Yes/no | 0.54 | 0.33–0.87 | **0.01** | 0.63 | 0.37–1.02 | 0.06 |
| Administration of platinum combination chemotherapy |  |  |  |  |  |  |
| Yes/no | 1.24 | 0.78–1.94 | 0.34 |  |  |  |
| Administration of TKI |  |  |  |  |  |  |
| Yes/no | 0.48 | 0.27–0.81 | **0.005** | 0.55 | 0.31–0.94 | **0.02** |
| Administration of ICIs |  |  |  |  |  |  |
| Yes/no | 0.78 | 0.39–1.42 | 0.44 |  |  |  |
| Recurrent pattern |  |  |  |  |  |  |
| Local recurrence/distant metastasis | 0.81 | 0.47–1.32 | 0.41 |  |  |  |
| Intracranial metastases at recurrence |  |  |  |  |  |  |
| Yes/no | 0.73 | 0.38–1.28 | 0.29 |  |  |  |
| Liver metastases at recurrence |  |  |  |  |  |  |
| Yes/no | 2.22 | 0.92–4.57 | 0.07 |  |  |  |
| Bone metastases at recurrence |  |  |  |  |  |  |
| Yes/no | 1.55 | 0.87–2.60 | 0.12 |  |  |  |
| Radiotherapy for postoperative lymph node recurrence |  |  |  |  |  |  |
| Yes/no | 0.67 | 0.36–1.17 | 0.17 |  |  |  |

HR, hazard ratio; CI, confidence interval; TKI, tyrosine kinase inhibitor; ICI, immune checkpoint inhibitor

*P*-values in bold are considered statistically significant (*p* < 0.05).
